# Near-infrared spectroscopy as a complementary age grading and species identification tool for African malaria vectors

**DOI:** 10.1186/1756-3305-3-49

**Published:** 2010-06-04

**Authors:** Maggy Sikulu, Gerry F Killeen, Leon E Hugo, Peter A Ryan, Kayla M Dowell, Robert A Wirtz, Sarah J Moore, Floyd E Dowell

**Affiliations:** 1 Griffith Medical Research College, a joint program of Griffith University and the Queensland Institute of Medical Research, Herston, QLD, 4006, Australia; 2 Ifakara Health Institute, Biomedical and Environmental Thematic Group, Ifakara and Dar es Salaam Branches, United Republic of Tanzania; 3 Liverpool School of Tropical Medicine and Hygiene, Vector Group, Liverpool, UK; 4 Centers for Disease Control and Prevention, Atlanta, Georgia, USA; 5 Disease Control and Vector Biology Unit, London School of Hygiene and Tropical Medicine, London, UK; 6 USDA ARS, Engineering and Wind Erosion Research Unit, Center for Grain and Animal Health Research, Manhattan, Kansas, USA

## Abstract

Near-infrared spectroscopy (NIRS) was recently applied to age-grade and differentiate laboratory reared *Anopheles gambiae sensu strico *and *Anopheles arabiensis *sibling species of *Anopheles gambiae sensu lato *complex. In this study, we report further on the accuracy of this tool for simultaneously estimating the age class and differentiating the morphologically indistinguishable *An. gambiae s.s*. and *An. arabiensis *from semi-field releases and wild populations. Nine different ages (1, 3, 5, 7, 9, 11, 12, 14, 16 d) of *An. arabiensis *and eight different ages (1, 3, 5, 7, 9, 10, 11, 12 d) of *An. gambiae s.s*. maintained in 250 × 60 × 40 cm cages within a semi-field large-cage system and 105 wild-caught female *An. gambiae s.l.*, were included in this study. NIRS classified female *An. arabiensis *and *An. gambiae s.s*. maintained in semi-field cages as <7 d old or ≥7 d old with 89% (n = 377) and 78% (n = 327) accuracy, respectively, and differentiated them with 89% (n = 704) accuracy. Wild caught *An. gambiae s.l*. were identified with 90% accuracy (n = 105) whereas their predicted ages were consistent with the expected mean chronological ages of the physiological age categories determined by dissections. These findings have importance for monitoring control programmes where reduction in the proportion of older mosquitoes that have the ability to transmit malaria is an important outcome.

## Findings

Vector survival is recognised as one of the most imperative determinants of vector-borne pathogen transmission. For example, malaria vectors can only transmit malaria parasites when they are at least 10 days old because of the lengthy period required for *Plasmodium *parasite development in the mosquito [1]. Traditionally, scientists relied upon observations of morphological changes in the reproductive system of female mosquitoes to estimate their physiological age [2-6] and to assess disease transmission potential [7,8]. However, these well-established age measurement techniques are labour intensive and they engage highly skilled personnel. These disadvantages render the techniques unsuitable for assessing age distribution at an operational level in large scale, community-randomized trials. New tools are therefore required that can effectively, rapidly, and accurately assess the ages of large numbers of mosquitoes as existing priority intervention technologies are scaled up [9,10] and new complementary approaches are developed and evaluated.

Furthermore, while accurate polymerase chain reaction methods do exist for differentiating sibling species such as those from the *An. gambiae *and *Anopheles funestus *complex in Africa [11-13], these methods are also somewhat laborious and expensive, limiting the numbers of mosquitoes which can be rigorously classified in most field studies. A convenient high throughput technique for simultaneously classifying and estimating the age of large numbers of mosquitoes would therefore enable biodemographic surveys of vector populations, and the impact of specific interventions upon them, on unprecedented scales.

Near-infrared spectroscopy (NIRS) uses the near-infrared region of the electromagnetic spectrum to quantitatively measure organic compounds e.g. O-H, N-H and C-O functional groups in biological samples. The spectrum collected is a result of the near-infra-red energy absorbed by a sample and is proportional to the amount of these functional groups present in samples. It is expected that a unique spectrum would be obtained for different age classes as well as different species of mosquitoes since it has been demonstrated that cuticular hydrocarbons change with age of mosquitoes [14] and that *An. arabiensis *have more water content in their body than *An. gambiae s.s*. [15]. After calibrations have been developed, the technique is very simple, requiring very little training or expertise. Whole insects are placed below a fibre-optic probe, a spectrum collected, and the age and species predicted from stored calibrations. Advantages of this technique are that insects can be scanned non-destructively, no sample preparation is required, and results are obtained in a few seconds.

NIRS was recently applied successfully to age and distinguish laboratory reared *An. gambiae s.s*. from *An. arabiensis *[16]. However, this study showed that additional data were needed to further develop calibrations and that additional field validation was needed. Herein we report on evaluation of the accuracy of this NIRS technique to estimate the ages and classify semi-field-maintained members of the *An. gambiae s.l*. complex, namely *An. gambiae s.s*. and *An. arabiensis*. We also further validated the accuracy of NIRS for differentiating wild caught *An. gambiae s.s*. from *An. arabiensis *and made a preliminary assessment of whether it might be useful for age grading wild-caught *An. gambiae s.l.*

*An. gambiae s.s*. (colony established in 1996 from Njage village, Kilombero, Tanzania) and *An. arabiensis *(colony established in 2007 from Sagamaganga village, Kilombero, Tanzania) were reared in the semi-field system established in the Ifakara Health Institute [17]. To avoid variance in adult emergence rates and development characteristics arising from environmental differences, larvae and pupae of *An. gambiae s.s*. and *An. arabiensis *were reared under their usual rearing conditions in their respective laboratory and semi-field colonies. Pupae of both siblings were transferred into small cages measuring 40 × 40 × 30 cm for emergence. Adult mosquitoes were moved to larger netted cages measuring 250 × 60 × 40 cm at day 0 (within a day of emergence). Adult cages for both sibling species were maintained within the semi-field system. A total of four cages for each species, representing four different age cohorts were reared. In addition to paper cups lined with moist filter papers as oviposition sites, two clay pots lined with a black cloth were positioned inside each cage to provide cool resting sites for these mosquitoes. Adult females were blood-fed twice a week by inserting a human (volunteer) arm inside the cage each time for 15 minutes (Ethical clearance No. IHRDC/EC4/CL.N96/2004) and provided with fresh 10% glucose solution daily. Wild mosquitoes were collected from Njage village, Kilombero, Tanzania using CDC-Light traps over two consecutive nights.

Prior to scanning, mosquitoes were anesthetised using chloroform. At least 40 females of each sibling species in each age cohort were scanned using an ASD Lab Spec 5000 (Boulder, Colorado) NIR spectrometer. Nine ages (1, 3, 5, 7, 9, 11, 12, 14, 16 d) of *An. arabiensis *and eight ages (1, 3, 5, 7, 9, 10, 11, 12 d) of *An. gambiae s.s*. from the semi field system were scanned. 105 wild caught *An. gambiae s.l*. were scanned for age and the sibling species identified as soon as they were collected from the field.

The scanning protocol has previously been described elsewhere [16]. Soon after the NIRS scans, 27% (n = 28) of the wild *An. gambiae s.l*. were dissected to determine their parity and ovarian development status [5]. Polymerase Chain Reaction was used to validate the accuracy of NIRS for differentiating wild *An. arabiensis *from *An. gambiae s.s*. [18].

A calibration model developed from partial least squares regression cross-validation [16] was used to predict the age and differentiate semi-field reared and wild caught *An. gambiae s.l*. sibling species. This was achieved by using the semi-field reared data in the calibration models. The improved species identification model included the following ages: 1, 5, 7, 11, 12 d for *An. arabiensis *and. 1, 3, 5, 9, 10 d *for An. gambiae s.s*. For differentiating species, *An. arabiensis *was assigned a value of "1", and *An. gambiae s.s*. assigned a value of "2". Samples were then classed depending on whether they were predicted above or below 1.5 classification cut off point.

**Figure 1:**
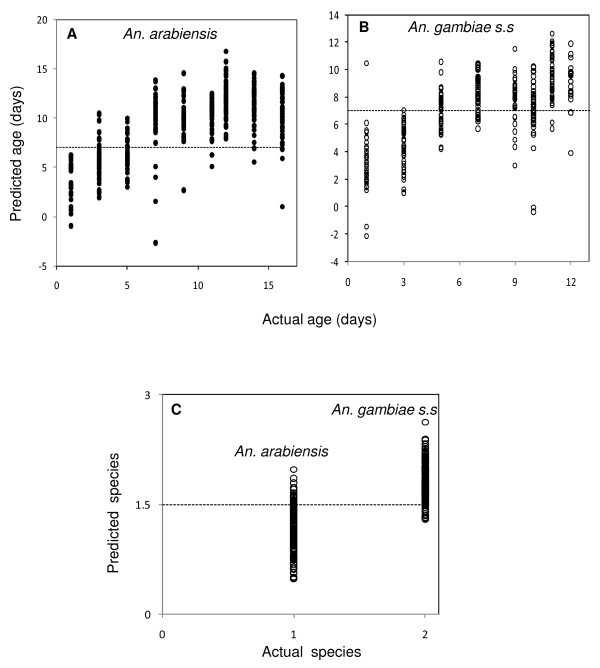
**NIRS age and species prediction for semi-field reared *An. arabiensis *and *An. gambiae s.s*. as determined from a cross-validation**. Panel A (*An. arabiensis*) and B (*An. gambiae s.s.*) indicate the actual age against the predicted age. The horizontal dotted line in Panel A and B separates young mosquitoes (<7 d) from old ones (≥7 d). Panel C shows the actual species (1 = *An. arabiensis *and 2 = *An. gambiae s.s.*) against the predicted species for 1, 5, 7, 11, 12 days old *An. arabiensis *and 1, 3, 5, 9, 10 days old *An. gambiae s.s*. The dotted line in panel C is the classification cut off point for the two sibling species (<1.5 for *An. arabiensis *and >1.5 for *An. gambiae s.s.*).

All mosquitoes that were identified as <7 or ≥7 days old by NIRS were classified as young or old, respectively. Out of the 704 *An. gambiae s.s*. and *An. arabiensis *reared under the semi-field conditions, 84% of them were accurately predicted as young or old (Figure 1A and 1B). Also, 89% (n = 377) of the *An. arabiensis *(Figure 1A) and 78% (n = 327) of the *An. gambiae s.s*. (Figure 1B) were accurately ranked as young or old.

The cross-validation technique distinguished the two sibling species reared in the semi-field system with 90% accuracy (Figure 1C) and the rest of the species categories that were not included in the initial calibration model (*An. arabiensis *3, 9, 14 d and *An. gambiae s.s*. 7, 11, 12 d) were classified with 88% accuracy.

**Figure 2:**
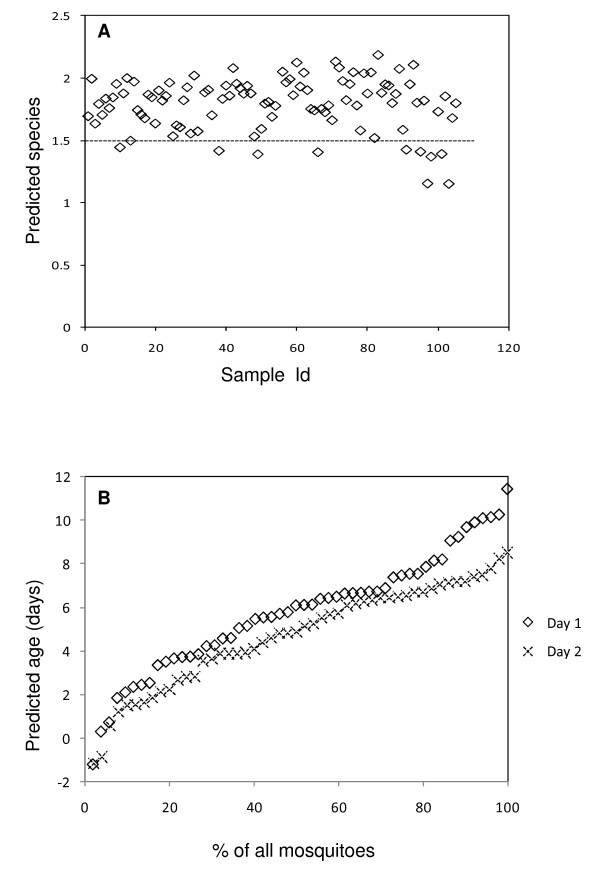
**NIRS age and species predictions for wild-caught mosquitoes**. Panel A indicates that 92% of all the wild mosquitoes were *An. gambiae s.s*. as predicted by NIRS. The dotted line in panel A is the classification cut off point for *An. gambiae s.s*. and *An. arabiensis *as predicted by NIRS (<1.5 for *An. arabiensis *and >1.5 for *An. gambiae s.s.*). Panel B shows the predicted cumulative age structure of wild mosquitoes from Njage collected in two consecutive nights.

For the wild specimens, NIRS predictions illustrated that 92% of the 105 *Anopheles *collected over the two sampling nights were *An. gambiae s.s*. (Figure 2A). To validate the results, 103 female *An. gambiae s.l*. were analyzed by PCR. An amplification success rate of 83% was obtained. PCR confirmed that NIRS had predicted the two sibling species with 90% accuracy. All the specimens predicted by NIRS to be *An. gambiae s.s*. were confirmed by PCR as correctly identified with the exception of those that could not be determined by PCR. Moreover, all the successful amplifications obtained were determined as *An. gambiae s.s*. while 2 of the PCR undetermined samples had been identified as *An. arabiensis *by NIRS. However, those predicted as *An. arabiensis *(10%) by NIRS but determined as *An. gambiae s.s*. by PCR, were very close to the 1.5 classification cut off point of the two sibling species. The confidence level associated with the NIRS classifications could be further increased by excluding any samples predicted as close to 1.5. The user would then have greater confidence that the remaining samples were correctly classified. A summary of the accuracy of age and species predictions for semi-field and wild *An. arabiensis *and *An. gambiae s.s*. by the NIRS is provided in table 1.

**Table 1:** The accuracy of NIRS for predicting the age and species of semi-field and wild *An. arabiensis *and *An. gambiae s.s*.

**Condition**	***An. arabiensis***				***An. gambiae s.s.***			
		
	**Age**		**Species ID**		**Age**		**Species ID**	
				
	**No. scanned**	**%correct**	**No. scanned**	**%correct**	**No. scanned**	**%correct**	**No. scanned**	**%correct**
								
Semi field	377	89	202	89*	327	78	201	91*
Wild	11	N/A**	11	N/A***	94	NA**	94	90

ID-Identification

No. -Number

*Accuracy for 1, 5, 7, 11 and 12 d *An. arabiensis *and 1, 3, 5, 9 and 10 d *An. gambiae s.s*. ages included in the previously developed model.

**Figure 3:**
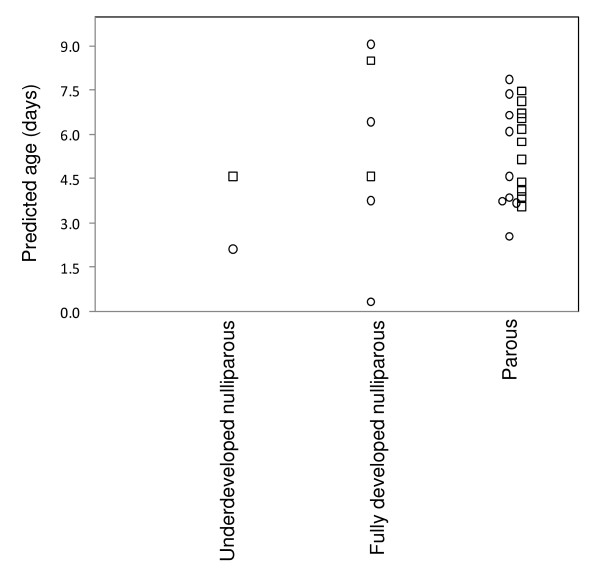
**NIRS age prediction of wild mosquitoes that were classified by parity dissections as underdeveloped (Christophers' stages ≤IIm) nulliparous, fully developed (Christophers' stages >IIm) nulliparous and parous for the first night (represented by circles) and the second night (represented by squares) **[5].

**Accuracy could only be verified on a proportion of the samples by parity dissections (see figure 3)

***Accuracy not verified since PCR was inconclusive on some samples and did not detect any *An. arabiensis *from the wild caught mosquitoes

NIRS age predictions for wild caught *An. gambiae s.l*. revealed a comparatively similar age structure for mosquitoes collected in two consecutive nights from the same houses (Figure 2B). Less than 22% of the mosquitoes collected each night were estimated as ≥7 days old. Figure 3 illustrates NIRS predicted ages of wild caught mosquitoes for which their ovary dissections were classified as underdeveloped (Christophers' stages ≤IIm) nulliparous, fully developed (Christophers' stages >IIm) nulliparous or parous for the first night (n = 13) and second night (n = 15) [5]. These results are consistent with mean chronological ages of nulliparous mosquitoes as determined previously by dissections [4,5]. However, the relatively low mean predicted age of parous mosquitoes relative to historical reports [4], is consistent with reduced adult female survivorship due to high insecticidal net coverage in this area (Russell et al., unpublished). These results also suggest that the population age structure from which these mosquitoes were collected from was quite stable for both nights.

Studies involving vector age assessments are crucial for determining the success or failure of any malaria control strategy that targets mosquito life span [19-22]. This study represents an extension of previous work [16] on laboratory and field specimens which indicated that additional data were needed to further develop calibrations and that further field validation was essential to verify the results. We have established and confirmed that NIRS can be applied to rapidly distinguish young from old *An. gambiae s.s*. and *An. arabiensis *and separate these morphologically similar species in areas where they do not occur sympatrically with other sibling species. Although NIRS was applied to age grade a relatively small sample of wild-caught specimens, insights were gained into the stability of the mosquito population age structure. Large-scale studies could potentially enable far more ambitious studies of malaria vector ecology, as well as the impact of control interventions upon vector bio-demography and transmission potential.

NIRS is non-destructive, rapid, and is associated with minimal sample processing costs after an initial outlay (approximately $40,000) for a NIR spectrometer. On the basis that NIRS can rapidly handle a large set of data with minimal labour and resources, the overall cost of using it on a large scale is dramatically reduced in the long run. Comparatively, the cost of cuticular hydrocarbon analysis depends largely on the accessibility of GC/FID or GC/MS instrumentation and, if the analysis is outsourced, can reach over US$50 per sample while the cost of transcriptional mosquito age grading has been estimated to be between $US7.5 to 10 per sample [23,24]. Additionally, current age assessment tools are not conducive to the rapid assessment of mosquito population age structure on a large scale while standard PCR [11] and multiplex PCR [12,13] for differentiating morphologically indistinguishable species is costly and time consuming. In terms of speed, NIRS was more than 10 times faster than parity dissections to determine physiological age. It required less than 15 seconds to scan one mosquito using NIRS and the spectrum obtained was analysed for both age and sibling species identification. Additionally, only one out of four technicians involved in this study could perform dissections to determine parity and these required approximately 3-5 minutes per mosquito. PCR reactions to determine sibling species required 2 people who took on average 5 hours working on 50 samples. Comparatively, it took less than 30 minutes to train 2 people to operate NIRS.

Although this study provides grounds for optimism, further work is clearly needed. Refinements to the method, such as the capacity to perform NIRS on preserved samples will practically facilitate large studies particularly in areas where field specimens must be preserved for future analysis. While there is a need for further age grading methods to determine population age structure on a finer scale, the value of NIRS is in the unique capacity to rapidly identify changes to mosquito population demography. Further studies are required to determine the capacity of this tool to differentiate and age grade other morphologically indistinguishable species in the *An. gambiae *complex and mosquitoes in other genera.

## Competing interests

All authors declare that they have no competing interests.

## Authors' contributions

MS drafted the manuscript; SJM, GFK, RAW, FED conceived the study; MS, LEH, PAR, SJM, FED and GFK designed the experiments; MS, FED, and KMD ran the experiments; FED analysed data; FED, GFK, SJM, LEH, PAR and RAW reviewed the manuscript. All authors read and approved the final manuscript.
